# Smoking and the Pathophysiology of Peripheral Artery Disease

**DOI:** 10.3389/fcvm.2021.704106

**Published:** 2021-08-27

**Authors:** Weiming Wang, Tingting Zhao, Kang Geng, Gang Yuan, Yue Chen, Youhua Xu

**Affiliations:** ^1^The State Key Laboratory of Quality Research in Chinese Medicine, Macau University of Science and Technology, Macau, China; ^2^Department of General Surgery (Vascular Surgery), The Affiliated Hospital of Southwest Medical University, Luzhou, China; ^3^Key Laboratory of Medical Electrophysiology, Ministry of Education and Medical Electrophysiological Key Laboratory of Sichuan Province, Institute of Cardiovascular Research, Southwest Medical University, Luzhou, China; ^4^Department of Nuclear Medicine, The Affiliated Hospital of Southwest Medical University, Luzhou, China

**Keywords:** smoking, peripheral artery disease, clinical evidence, molecular mechanism, review

## Abstract

Smoking is one of the most important preventable factors causing peripheral artery disease (PAD). The purpose of this review is to comprehensively analyze and summarize the pathogenesis and clinical characteristics of smoking in PAD based on existing clinical, *in vivo*, and *in vitro* studies. Extensive searches and literature reviews have shown that a large amount of data exists on the pathological process underlying the effects of cigarette smoke and its components on PAD through various mechanisms. Cigarette smoke extracts (CSE) induce endothelial cell dysfunction, smooth muscle cell remodeling and macrophage phenotypic transformation through multiple molecular mechanisms. These pathological changes are the molecular basis for the occurrence and development of peripheral vascular diseases. With few discussions on the topic, we will summarize recent insights into the effect of smoking on regulating PAD through multiple pathways and its possible pathogenic mechanism.

## Introduction

With changes in global population epidemiology, the incidence of PAD may increase in the future (1). In a broad sense, PAD includes arterial stenosis and occlusion caused by atherosclerotic plaques and thrombosis, arterial inflammation, arterial dilatation, peripheral artery dysfunction, external pressure lesions, etc., and its most strict definition refers to atherosclerotic occlusion disease (2, 3). The occurrence of peripheral vascular disease is closely related to traditional cardiovascular factors (smoking, hypertension, diabetes, and dyslipidemia) and the aging of the population (4). However, for some low- and middle-income countries, environmental factors such as poverty, industrialization, and infection may also affect the occurrence and development of peripheral vascular diseases. Smoking is a particularly strong risk factor for peripheral vascular disease, and with further research, existing studies have indicated that smoking independently correlates with peripheral vascular disease (1, 3).

### Clinical Evidence of Smoking-Associated PAD

Numerous studies have confirmed the link between active smoking and PAD. A systematic review showed that half of PAD cases are due to smoking (5). Compared with non-smokers, people who have ever smoked are still at higher risk of developing PAD (6, 7). Meanwhile, passive smoking also increases the risk of vascular damage or the diagnosis of PAD (8).

The harm caused by smoking cannot be underestimated, but researchers have not clearly determined whether quitting smoking effectively reduces the incidence of cardiovascular disease. Although quitting smoking reduces the risk of cardiovascular morbidity and mortality in patients with a history of smoking, the continuing effects of smoking are still relatively high. Therefore, smoking cessation is never too late to reduce the cardiovascular risk (9). At the same time, David Conen also confirmed that quitting smoking significantly reduces the risk of PAD, but the incidence of PAD is still increasing, even for those who quit smoking (10).

In terms of the effect of smoking on PAD after surgery, studies have shown that smoking intensity exerts an unusually large effect on the prognosis of patients with symptomatic lower extremity revascularization. Compared with patients who continue to smoke, the mortality rate of patients who quit smoking is lower and these patients have a higher survival rate without amputation (11, 12).

## Smoking and Atherosclerosis

### Clinical Evidence

Smoking has been widely regarded as the leading risk factor for clinical cardiovascular disease that directly affects atherosclerosis. Active smoking and passive smoking directly correlate with the occurrence of atherosclerosis (13, 14). Strict smoking bans have reduced the number of active smokers worldwide, but non-smokers may still be exposed to environmental smoke in their homes or workplaces through passive smoke inhalation. Therefore, passive smoking must be prevented and controlled (8). At the same time, in terms of passive smoking, children's long-term cigarette exposure is significantly correlated with the risk of developing severe carotid atherosclerotic plaques in early adulthood, which is ~2 times higher than those without exposure (15). A dose-response relationship has been observed between the time and amount of smoking and the occurrence of atherosclerosis. The only effective measure to reduce the risk of smoking-related atherosclerosis is to quit smoking. However, after quitting smoking, the continuous effects caused by smoking are not completely eliminated (16, 17).

**Figure 1:**
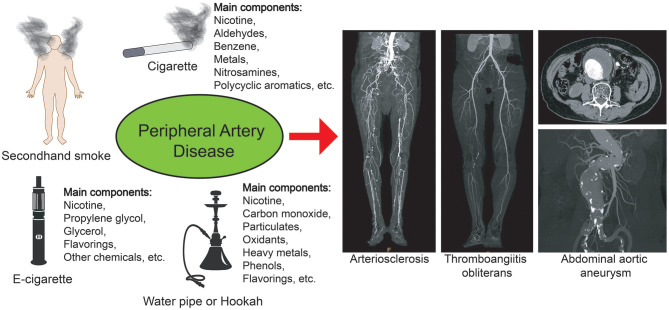
Smoking and peripheral artery disease. Different types of smoking may cause peripheral vascular diseases, such as arteriosclerosis, thromboangiitis obliterans, and abdominal aortic aneurysm. The ingredients in different cigarettes and substitutes are different, but they may all pose a risk of cardiovascular disease.

With the transformation of tobacco marketing methods, in addition to traditional smoking and its methods, some new types of tobacco substitutes (such as electronic cigarettes, hookahs, etc.) have gradually become new consumption methods and are more commonly used by young people (18, 19). Hundreds of different chemical components are present in various tobacco substitutes (20–22). Although qualitative and quantitative comparisons of the compositions of e-cigarettes, hookah aerosols and cigarette smoke have been performed, the risks of e-cigarettes and hookah aerosols are considered lower than those of cigarette use (22–24). Therefore, the current misconceptions about this new form of tobacco use and its potentially harmful health effects are gradually contributing to the risk of cardiovascular disease in this group of people. In summary, e-cigarettes, hookahs or cigarettes, as well as active or passive smoking, exert serious effects on cardiovascular disease in the population (Figure 1).

### Smoking-Related Molecular Mechanisms

Atherosclerosis is the primary pathological basis of PAD. Its main feature is the formation of atherosclerotic plaques, which are characterized by the accumulation of lipids and immune cells in the blood vessel wall covered by a collagen fiber cap. The abnormal metabolism and distribution of lipids play an important role in the occurrence and development of atherosclerosis (25, 26). Studies have shown that smoking affect the composition and distribution of blood lipids in ApoE-/- and LDLr-/- mice, accelerates the formation of aortic plaques and increases the total cholesterol content, thereby inducing the occurrence of atherosclerosis. Smoking cessation slows plaque progression and leads to decreased levels of many lipid types in the plasma and aorta (27–29). However, Kunimoto et al. reported that cigarette smoke had no effect on serum cholesterol and triglyceride levels in ApoE-/- mice (30). The author proposes that this difference may be due to a certain difference in the technology and composition of smoke exposure, which ultimately led to the ambiguity of the results of the study. Therefore, some researchers adopted the model of atherosclerosis induced by nicotine, which is the major addictive component of all components of cigarette smoke, to avoid the confounding effects of other components (31). A large body of evidence also supports the role of nicotine in promoting atherosclerosis (23, 32). However, due to the particular characteristics of nicotine, its acquisition requires strict approval procedures, and thus it is not widely used in basic research.

**Figure 2:**
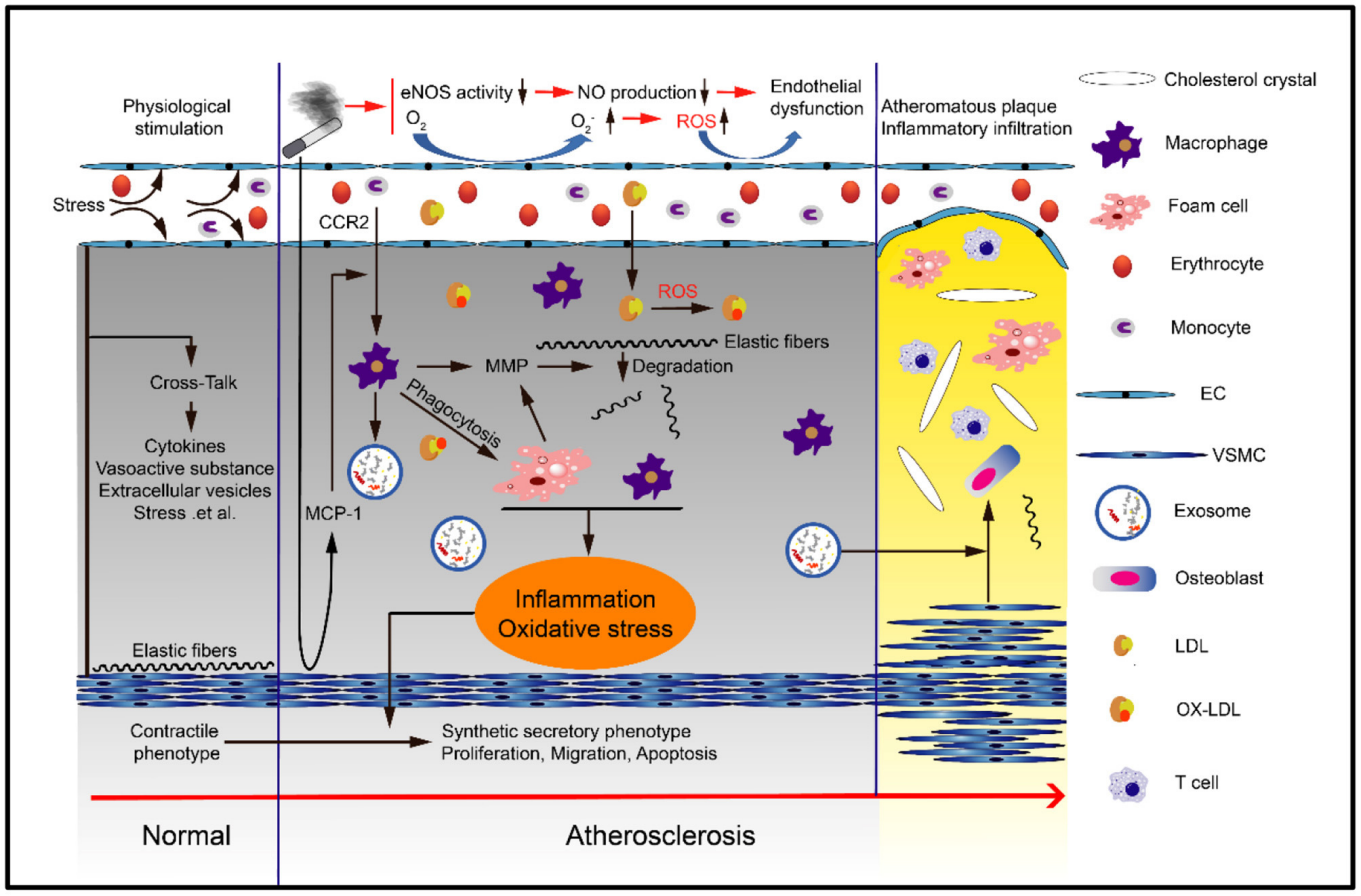
Smoking can cause atherosclerosis. Smoking can cause endothelial cell dysfunction, monocyte differentiation into macrophages and eventually foam cells, smooth muscle cell proliferation, migration and phenotypic transformation, and extracellular matrix degradation and remodeling. These processes are typically mediated by oxidative stress and inflammation.

In addition to abnormal lipid metabolism, various cells are also involved in the occurrence of atherosclerosis (33). At present, various studies have shown that for long-term smokers, whether they are active smokers or passive smokers, harmful substances may induce various phenotypic changes and dysfunction of macrophages, endothelial cells and smooth muscle cells through various mechanisms, thus promoting the occurrence and development of vascular diseases (34, 35) (Figure 2).

#### Smoking and Endothelial Cells

**Figure 3:**
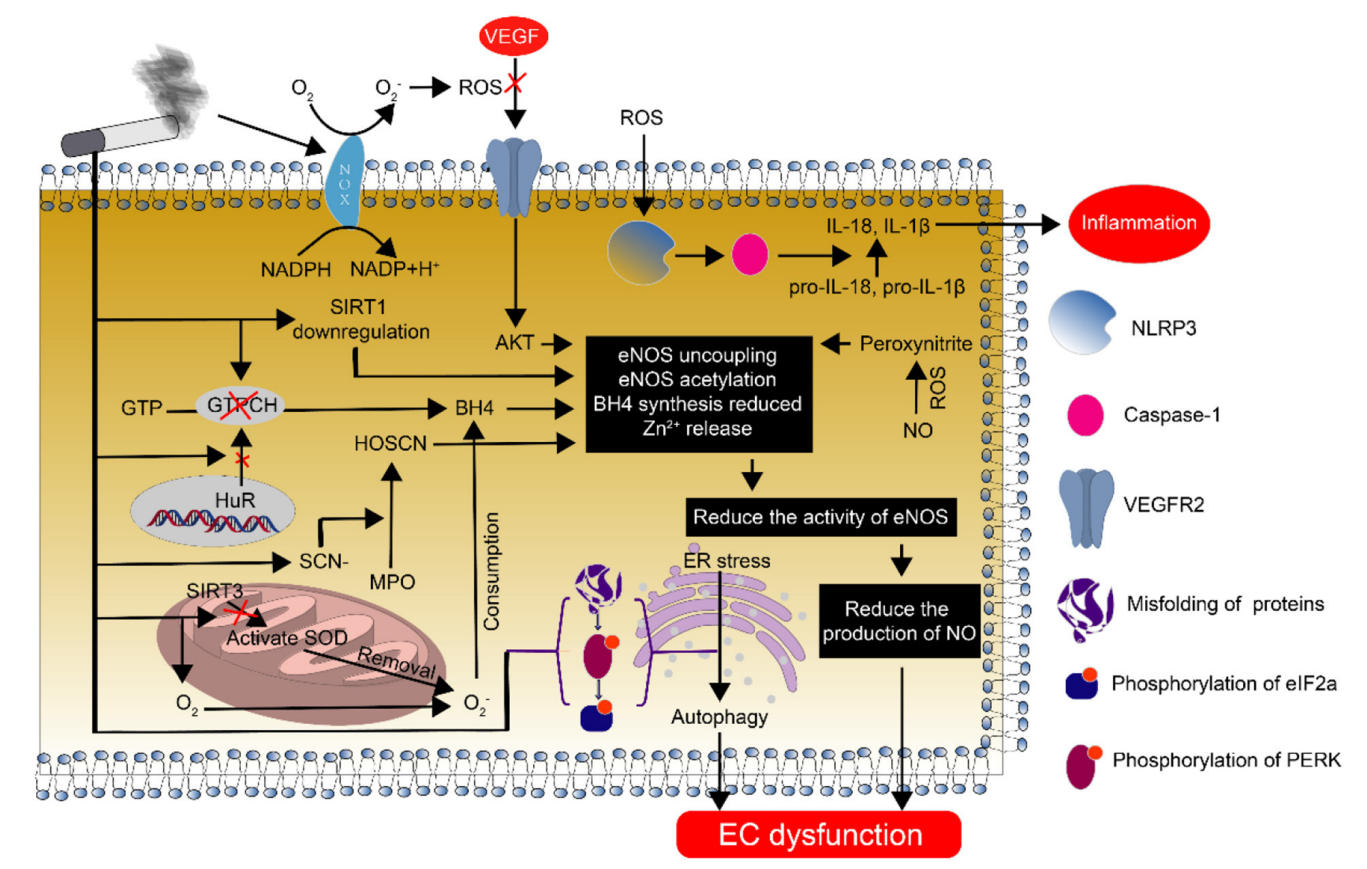
Endothelial cell dysfunction caused by smoking and its mechanism. Smoking causes endothelial cell dysfunction through various pathways. The most important pathway is the decrease in NO synthesis due to eNOS deficiency and the reduction in eNOS function, which plays an important regulatory role in endothelial dysfunction.

Endothelial dysfunction is one of the earliest abnormalities that is observed during the development of atherosclerosis, and endothelial nitric oxide synthase (eNOS) plays a crucial role in regulating endothelial dysfunction (36, 37) (Figure 3). The monomer eNOS has no activity. However, when monomeric eNOS forms a stable dimer through heme and BH4, it has biological activity. GTP cyclohydrolase (GTPCH) is an important rate-limiting enzyme in the synthesis of BH4. CSE reduces the synthesis of GTPCH by inhibiting the transport of HuR from the nucleus to the cytoplasm, thereby affecting the synthesis of BH4. Finally, it affects the activity of eNOS and reduces the production of nitric oxide (NO) (38–40). At the same time, CSE can also inhibit the synthesis of BH4 by reducing the expression of dihydrofolate reductase (DHFR), and thus affect the activity of eNOS (41). CSE increases the expression of thiocyanate (SCN-), which is used by myeloperoxidase (MPO) to form hypothiocyanic acid (HOSCN). HOSCN reduces the level of active dimerized eNOS and increases the release of inactive monomers and Zn^2+^ (42, 43). HOSCN-modified low-density lipoprotein also impairs eNOS activity, thereby reducing NO production (44). CSE-derived reactive oxygen/nitrogen species interact with VEGFR2, resulting in post-translational modifications of VEGFR2 that decrease the activity of the downstream Akt/eNOS/NO signaling pathway, leading to endothelial dysfunction (45).

Oxidative stress is the interaction between the body's response to oxidation caused by various pathological factors and antioxidants. Normally, it is in a balanced state. However, when the effect of oxidation is greater than the antioxidant effect, the infiltration of inflammatory cells occurs, the secretion of various proteases and cytokines increases, and a large amount of oxidative intermediate products are generated, which in turn cause a series of physiological and pathological changes (46, 47). Studies have confirmed that oxidative stress is also involved in CSE-induced endothelial cell dysfunction (48–50). Mitochondria are an important source of O${}_{2}^{-}$ free radicals, and excessive O${}_{2}^{-}$ accumulation induces endothelial cell dysfunction through multiple pathways. Under normal physiological conditions, the removal of O${}_{2}^{-}$ mainly occurs through the antioxidant SOD, and SIRT3 is indispensable for the regulation of SOD activation and mitochondrial metabolism in mitochondria (51). CSE reduces the expression of the mitochondrial deacetylase SIRT3, thereby increasing SOD hyperacetylation, subsequently hindering O${}_{2}^{-}$ clearance and resulting in its excessive accumulation in the body (52). Meanwhile, thiol-stabilized compounds in CSE activate NADPH oxidase and increase the production of endothelial O${}_{2}^{-}$ (53). Increased O${}_{2}^{-}$ production consumes the cofactor tetrahydrobiopterin necessary for eNOS activity (39). CSE-mediated oxidative stress downregulates SIRT1 (it activates eNOS by reducing the acetylation of eNOS), causing increased eNOS acetylation and thereby reducing NO-mediated signaling and endothelial dysfunction (54).

The production of a large amount of ROS leads to the rapid inactivation of NO and the formation of peroxynitrite, thereby reducing the biological activity of NO (23). Peroxynitrite also destabilizes the eNOS dimer by detaching BH4 from the active site of the zinc finger complex (55–57). At the same time, peroxynitrite is also a strong biological oxidant that promotes post-translational modification of proteins (including the eNOS protein), alters metabolic pathways, and ultimately leads to increased superoxide production and reduced NO production (58, 59). CSE results in reduced eNOS protein quality, decreased mRNA expression, and inhibited calpain activity, resulting in impaired irreversible inhibition of the activity of eNOS and a reduced content in endothelial cells, which ultimately impairs endothelium-dependent vasodilation and decreases NO synthesis (60, 61). In addition, CSE activates the NLRP3 inflammasome through ROS, causing the release of downstream IL-1β and IL-18 and other inflammatory factors, leading to functional changes such as autophagy, pyrolysis, and apoptosis of endothelial cells (56, 62, 63).

Studies have shown that CSE can cause vascular wall contraction by activating Rho kinase (64, 65). On the one hand, activated Rho kinase directly phosphorylates eNOS (Thr113), thereby inhibiting NO production in endothelial cells (66, 67). On the other hand, it can promote the contraction of smooth muscle cells by regulating the sensitivity of calcium ions (68, 69). At the same time, CSE can also cause the activation of Rho kinase in macrophages, and then participate in the occurrence and development of atherosclerosis (70, 71).

According to clinical studies, plasma levels of asymmetric dimethylarginine (ADMA) are significantly higher in smoking patients than in non-smokers (72). ADMA, a metabolite of L-arginine, is an endogenous inhibitor of eNOS (73). Studies have further confirmed that cigarette extract not only inhibits L-arginine uptake but also reduces the expression of L-arginine transporter CAT1, thereby reducing NO production (74). However, L-arginine supplementation improves arterial diastolic function in healthy smokers (75).

**Figure 4:**
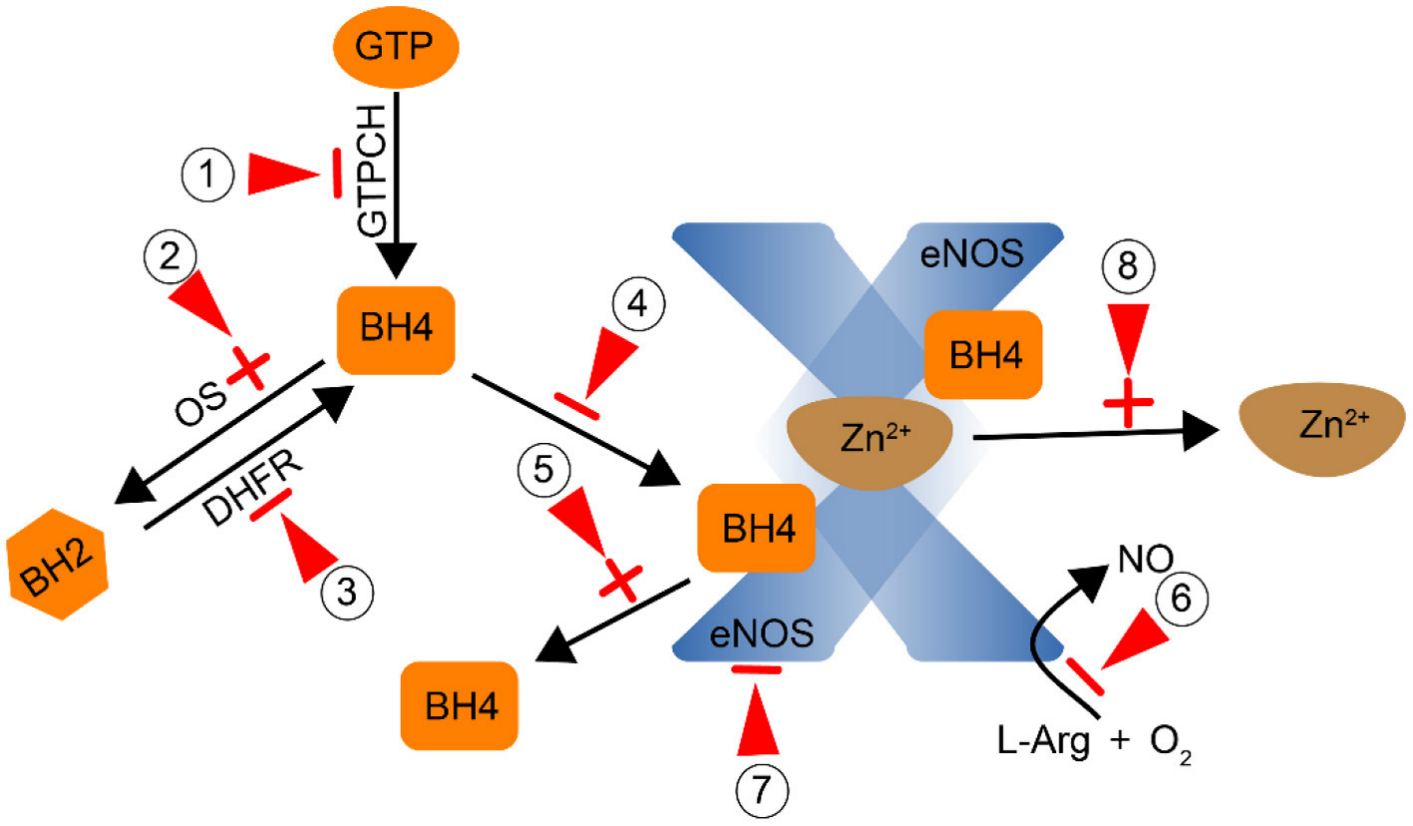
Smoking affects the activity of eNOS by direct or indirect action (Red arrow: point of action). Inhibit GTPCH and reduce the synthesis of BH4; Promote the conversion of BH4 to BH2 through oxidative stress; Inhibit DHFR and reduce the conversion of BH2 to BH4;, ⑤ Prevent the combination of BH4 and eNOS to form a stable dimer; ⑥ Inhibit the conversion of L-Arg to NO; ⑦ Inhibit the activity of eNOS; ⑧ Promote the dissociation of Zn^2+^ from the dimer.

*In vitro* studies have shown that CSE reduces protein synthesis by inducing protein misfolding in the endoplasmic reticulum. At the same time, a large number of unfolded proteins interact with each other and lead to the phosphorylation of PERK and eIF2a, which will prolong endoplasmic reticulum stress and activate autophagy-related programs, resulting in endothelial cell dysfunction (76). In other organelles, CSE induces mitochondrial damage and changes in the membrane potential by activating the NF-KB and NLRP3 signaling pathways. However, this process is reversed by MitoQ (77). In addition, cigarette extracts also upregulate the expression of galectin-3, thereby increasing the levels of p-AMPK and decreasing the levels of p-mTOR, resulting in increased autophagy in endothelial cells. At the same time, cigarette extract increases ROS production and downregulates eNOS expression, thereby affecting the tube formation and migration ability of endothelial cells (78). At present, there are many studies on smoking and endothelial cells, but most of them focus on endothelial dysfunction (Figures 3, 4).

#### Smoking and Monocytes-Macrophages

Circulating monocytes are derived from hematopoietic progenitor cells in the bone marrow. According to the expression levels of CD14 and FCγRIII immunoglobulin receptor CD16, which are components of the lipopolysaccharide receptor complex, three subgroups of monocytes have been identified in humans, including CD14++ CD16–, CD14+ CD16+, and CD14+ CD16++ monocytes. The activation of monocytes participates in the formation of PAD (79, 80). Cigarettes induce the production of various chemokines and proinflammatory cytokines. These factors promote the adhesion of monocytes to endothelial cells, drive monocytes to enter the endothelium, and undergo phenotypic changes. The most important regulatory factor is CC chemokine receptor 2 (CCR2) and its ligands monocyte chemoattractant protein 1 (MCP-1) and monocyte chemoattractant protein 3 (MCP-3) (81–83). Second, the particulate components of CSE activate protein kinase C (PKC), resulting in increased expression of the peripheral blood monocyte adhesion ligand CD11b and endothelial cell counter receptors VCAM1, ICAM-1 and ELAM-1. The expression of these ligands and counter receptors leads to enhanced adhesion of monocytes to endothelial cells, which is a factor initiating the pathogenesis of CSE and causing inflammation in blood vessel walls (84, 85).

Aryl hydrocarbon receptor (AHR) is a member of the transcriptional regulator bHLH (basic helix-loop-helix)-PAS (Per-ARNT-Sim) family, which mainly regulates various developmental and physiological functions (86, 87). Normally, AHR forms a dormant complex with HSP90, XAP2, and p23 in the cytoplasm (88). When exposed to smoke for a long time, the polycyclic aromatic hydrocarbon (PAH) ligand in cigarette smoke binds to AHR and causes a conformational change (89). The continuously activated AHR may promote the occurrence of atherosclerosis (90–92). At the same time, AHR can induce the production of AHRR (AHR repressor) and then regulate the activation of AHR through negative feedback. However, cigarette smoke continuously activates the AhR signaling pathway, which potentially drives changes in the methylation of the AHRR gene, accompanied by histone modification and enhancer activation. Methylated AhRR will undergo regional DNA conformation changes that affect the interaction of protein factors and DNA, subsequently altering gene transcription and expression and participating in the regulatory process of atherosclerosis (93–95). After a conformational change in AHR, HSP90 is released from the complex. Studies have shown that HSP90 interacts with Akt to mediate the phosphorylation of eNOS (96–98).

Monocytes and macrophages that enter the endothelium release large amounts of MPO that forms hypochlorous acid (HOCl) and hypothiocyanic acid (HOSCN). Macrophages exposed to HOSCN exhibit increased release of inflammatory cytokines and chemokines, including MCP-1, TNF-α, and IL6, and the subsequent of amplification of the inflammatory cascade. However, HOSCN-induced cytokine/chemokine expression and cell death are reduced by inhibiting the NF-kB signaling pathway (99). Further research showed the differential expression of miRNAs in smokers and non-smokers. This differential expression also participates in the regulation of monocyte phenotypic changes. The expression of miR-124-3p in smokers is significantly higher than that in non-smokers, and changes in surface markers are accompanied by changes in the monocyte phenotype. When miR-124-3p is overexpressed, the expression of CD206 on the surface of monocytes is significantly upregulated (100).

#### Smoking and Vascular Smooth Muscle Cells

Under normal physiological conditions, the relaxation and contraction of smooth muscle cells in the arterial wall is necessary to maintain relatively stable blood flow and blood pressure. However, when cells are stimulated with pathological factors, smooth muscle cell proliferation, migration, apoptosis and phenotypic changes are crucial in the pathological process of atherosclerosis (101). Nicotinic acetylcholine receptors (nAChRs) are members of the superfamily of cys-loop ligand-gated ion channels (cysLGICs). They are widely distributed transmembrane proteins in vascular smooth muscle cells and are involved in regulating ion homeostasis and maintaining functional changes in blood vessels (102–104). Nicotine in cigarette smoke is an important ligand for the α1 nicotinic acetylcholine receptor, which directly induces the transformation of VSMCs from a contractile phenotype to a synthetic phenotype through the nicotinic acetylcholine receptor and G protein-coupled receptor. It also promotes the migration of VSMCs from the media to atheromatous plaques in the vascular intima (105). At the same time, the gated ion channel is open, resulting in the influx of a large amount of Ca^2+^. When the concentration of Ca^2+^ in the cytoplasm is further increased, it will cause a conformational change in calpain-1 that triggers its proteolytic activity. Therefore, calpain promotes remodeling of the actin cytoskeleton in VSMCs to form podosomes by activating PKC, accompanied by the degradation of extracellular matrix (ECM) (106). On the other hand, the extracellular matrix is degraded by activated MMP-2/MMP-9 (107, 108). Second, an increased influx of Ca^2+^ activates the ERK1/2-Egr-1 signaling pathway and form a cascade amplification effect, thereby participating in nicotine-induced smooth muscle cell proliferation (109).

Nicotine also participates in the functional and phenotypic changes of smooth muscle cells through nAChR-induced regulation of multiple related downstream signaling pathways. Knockdown of nAChRα1 significantly reduces the CSE-induced phosphorylation of STAT3, Akt, and mTOR, while overexpression of nAChRα1 exerts the opposite effect. STAT3 directly interacts with the nicotine receptor nAChRα1 to regulate the nuclear translocation of STAT3 and its binding to the Akt promoter region, thereby promoting the proliferation and migration of VSMCs and inflammation mediated by macrophages, which in turn causes atherosclerosis. STAT3 inhibition significantly reduces nicotine-induced atherosclerosis (110). Nicotine also modulates the release of vascular endothelial growth factor (VEGF) in VSMCs in rats, and this process is blocked by selective inhibitors of epidermal growth factor receptor (EGFR) kinase and MEK, leading to reduced ERK phosphorylation and decreased VEGF release. Therefore, nicotine promotes VEGF release from VSMCs by activating the EGFR-ERK pathway and increases the risk of cardiovascular disease in smokers (111). In addition, nicotine causes oxidative stress to increase the release of inflammatory bodies through nAChRs and promote smooth muscle cell autophagy through the NF-κB signaling pathway, which in turn causes smooth muscle cell phenotypic transformation (112, 113).

Endothelin plays an important role in maintaining circulatory homeostasis (114). Studies have shown that cigarette smoke or nicotine can affect the expression of ETA (mainly distributed in the smooth muscle cells of the blood vessel wall) and ETB (mainly distributed in the endothelial cells of the blood vessel wall) receptor expression, thereby regulating the vasomotor reactivity (115–117). Cigarette smoke exposure can promote the upregulation of ETA receptor expression through MEK1/2 (118), ERK1/2 (119), MAPK (120), and NF-kB pathways (121), and thus induce the contraction of smooth muscle cells dependent on Ca^2+^ signaling channels (122, 123). At the same time, cigarette smoke can also increase the expression of ETB receptor in endothelial cells, and cause endothelial dysfunction and contraction of VSMCs through the effect of ET-1 and the reduction of NO synthesis (66, 121, 124).

CSE or nicotine promotes bFGF production in smooth muscle cells, which subsequently promotes the mitosis of cells, accompanied by the phosphorylation of ERK and c-Jun and increased expression of cyclinD1. When ERK phosphorylation is inhibited, the levels of p-c-Jun and cyclinD1 decrease correspondingly, along with a decrease in cell viability. When c-Jun is overexpressed or knocked down, the expression of cyclinD1 is also affected accordingly. CSE promotes smooth muscle cell proliferation through the p-ERK-p-c-Jun-cyclin D1 pathway (125, 126).

**Figure 5:**
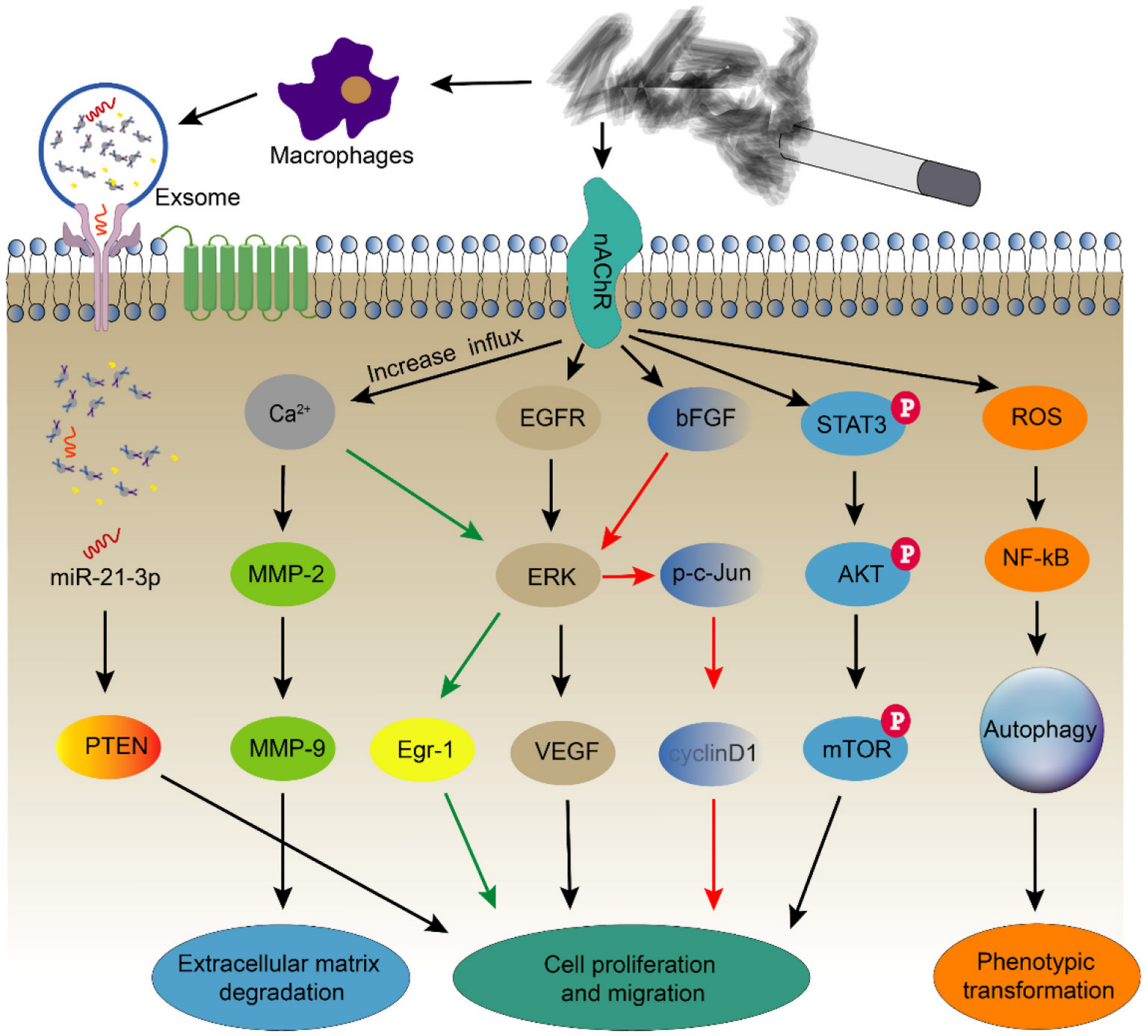
Smoking alters the function and phenotype of smooth muscle cells. Smoking induces smooth muscle cell autophagy through oxidative stress and causes smooth muscle cell proliferation and migration through multiple signaling pathways. At the same time, it also regulates changes in the functions of smooth muscle cells by stimulating macrophages to secrete exosomes.

In addition, smoking reduces acetylcholine (ACh)-induced vasodilation. This mechanism may be caused by the hyperpolarization of VSMCs induced by stimulating endothelial cells to reduce the production of NO and PGI2 (127). Based on recent studies, the functional and phenotypic changes in smooth muscle cells play a vital role in the onset and progression of atherosclerosis (Figure 5), but the specific mechanism by which smoking regulates smooth muscle cells still requires further research.

### Smoking and Animal Models of Atherosclerosis

Smoking can regulate ECs, VSMCs and mononuclear macrophages through a variety of mechanisms to participate in the occurrence and development of atherosclerosis (Table 1). However, *in vivo* experiments, many studies have also confirmed the relationship between smoking and atherosclerosis in animals (138, 139). Currently, the animal models used to study atherosclerosis mainly include ApoE-/- mice, LDLR-/- mice, and ApoE-/- and LDLR-/- mice. Models have also been derived from different species, such as mice, rabbits, pigs and non-human primates (140). However, different animal models (focus on comparing rabbits and mice) have different advantages and limitations (140–148) (Table 2).

**Table 1:** Smoking and various types of cell function changes, phenotypic transitions and their molecular mechanisms.

**Cell type/plasma**	**Cigarette ingredients**	**Function changes/phenotypic transitions**	**Molecular mechanism**	**Disease**	**References**
ECs	CSE	Dysfunction	Inhibit the transportation of HuR from the nucleus to the cytoplasm, and reduce the synthesis of GTPCH, thereby reducing the synthesis of BH4, to ultimately reduce the activity of eNOS and the production of NO	ASO	(38–40)
	CSE	Dysfunction	Reduce the expression of DHFR, inhibit the synthesis of BH4, and then reduce the activity of eNOS.	ASO	(41)
	CSE	Dysfunction	Promote the increase of thiocyanate (SCN-) expression, thereby promoting the increase of HOSCN synthesis and reducing the activity of eNOS and the production of NO	ASO	(42–44)
	CSE	Dysfunction	Reactive oxygen/nitrogen interacts with VEGFR2, leading to post-translational modification, thereby inhibiting the downstream Akt/eNOS/NO signaling pathway	ASO	(45)
	CSE	Dysfunction	Decrease the expression of SIRT3 and increase the over-acetylation of SOD, which leads to the obstruction of O_2_ clearance and excessive accumulation	ASO	(51, 52)
	CSE	Dysfunction	Activate NADPH oxidase and increase the production of endothelial O^2−^. The increased O^2−^ will consume tetrahydrobiopterin, a cofactor required by eNOS	ASO	(39, 53)
	CSE	Dysfunction	Downregulate SIRT1, promote the increase of eNOS acetylation, thereby reducing the production of NO	ASO	(54)
	CSE	Dysfunction	The production of ROS will lead to the rapid inactivation of NO and the formation of peroxynitrite : Reducing the biological activity of NO; Separating BH4 and destroying the stability of eNOS; Promoting the modification of protein (eNOS) and changing the metabolic pathway	ASO	(23, 55–59)
	CSE	Dysfunction	Inhibit the uptake of arginine and reduce the expression of arginine transporter CAT1, thereby reducing the synthesis of NO	ASO	(74)
	CSE	Dysfunction	Misfolded proteins reduce protein (eNOS) synthesis. At the same time, the interaction of unfolded proteins leads to phosphorylation of PERK and eIF2a, prolonging endoplasmic reticulum stress and activates autophagy	ASO	(76)
	CSE	Dysfunction	Activate NF-kB and NLRP3 signaling pathways to promote mitochondrial damage, leading to energy disorders	ASO	(56, 62, 63, 77)
	CSE	Dysfunction	Upregulate the expression of galectin-3, thereby promoting the upregulation of p-AMPK and down-regulation of p-mTOR, leading to increased autophagy expression	ASO	(78)
	CSE	Dysfunction	Activate Rho kinase and promote phosphorylation of eNOS (Thr113), thereby inhibiting the production of NO in ECs	ASO	(66, 67)
	CSE	Dysfunction	Increased expression of ETB receptors causes endothelial dysfunction through the action of ET-1	ASO	(121, 124)
	CSE	Arterial-spasm thrombosis	Interact with eNOS mutated gene locus to reduce the production of vasodilatory NO	TAO	(128)
Monocyte-macrophages	CSE	Inflammation	Produce various chemokines and pro-inflammatory cytokines (CCR2, MCP-1, MCP-3), thereby increasing the adhesion of monocytes to the blood vessel wall	ASO	(81–83)
	CSE	Inflammation	Activation of PKC leads to increased expression of CD11b and VCAM1, ICAM-1 and ELAM-1, thereby increasing the adhesion of monocytes to the vessel wall	ASO	(84, 85)
	PAH	Affect gene transcription and expression	Activate the AHR signaling pathway and drive the methylation of the AHRR gene to change the DNA conformation and affect the interaction between protein factors and DNA	ASO	(93–95)
	CSE	Cell transformation	Activate the RANKL-RANK pathway to promote the transformation of macrophages into osteoclasts	AAA	(129)
	CSE	Extracellular matrix degradation	Upregulate NFATc1, promote macrophage activation, and promote the expression of osteoclast production related proteases TRAP, cathepsin K and MMP-9	AAA	(130, 131)
	CSE	Osteoclastogenesis	Activate macrophages through NF-kB, increasing the expression of MCP-1, MMP-9, IL-8 and TNF-α	AAA	(132, 133)
	CSE	Tissue remodeling and inflammation	Increased expression of MMP-9 and MMP-2: Activating TGF-β; Inactivating protein-1α by cleaving serpins at an inhibitory site region, which will induce the expression of proinflammatory molecules	AAA	(134, 135)
VSMCs	CSE	Contraction	Activate Rho kinase and regulate the sensitivity of calcium ions	ASO	(68, 69)
	Nicotine	Phenotypic transitions	Activate nAChRs and GPCRs to promote the conversion of contractile type to synthetic type	ASO	(105)
	Nicotine	Cytoskeleton remodelingMatrix degradationCell Proliferation	Increase calcium influx: Changing calpain-1 conformation, then activating PKC to promote actin morphology changes; Activating MMP-2/MMP-9 signaling pathway; Activating ERK1/2-Egr−1 signal pathway	ASO	(106–109)
	Nicotine	Proliferation and migration	Promote the phosphorylation of STAT3, Akt, and mTOR through the AChRα1 receptor	ASO	(110)
	Nicotine	Proliferation and migration	Activate the EGFR-ERK pathway and promote the release of VEGF	ASO	(111)
	Nicotine	Phenotypic transitions	Cause oxidative stress, which activates the NF-kB signaling pathway, promotes autophagy and the transition of VSMCs from contractile to synthetic	ASO	(112, 113)
	CSE	Contraction	Activate MEK1/2, ERK1/2, MAPK, NF-kB, thereby promoting the upregulation of ETA receptor expression	ASO	(118–123)
	CSE/ Nicotine	Proliferation	Promote the production of bFGF, which in turn activates the p-ERK-p-c-Jun-cyclinD1 pathway	ASO	(125, 126)
	CSE	Contraction	Reduce the production of NO and PGI2 in ECs, thereby promoting hyperpolarization of VSMCs	ASO	(127)
Plasma	CSE	Inflammation	Increase expression of MMP-9 and HMGB-1	TAO	(136, 137)

*CSE, Cigarette smoke extract; VSMCs, Vascular smooth muscle cells; ECs, Endothelial cells; DHFR, Dihydrofolate reductase; PAH, Polycyclic aromatic hydrocarbons; GTPCH, GTP cyclohydrolase; eNOS, Endothelial nitric oxide synthase; NO, Nitric oxide; ROS, Reactive oxygen species; VEGF, Vascular endothelial growth factor; VEGFR2, Vascular Endothelial Growth Factor Receptor 2; BH4, Tetrahydrobiopterin; PKC, Protein kinase C; TGF-β,Transforming growth factor-β; MMP, Matrix metalloproteinase; nAChRs, Nicotinic acetylcholine receptors; GPCRs, G protein-coupled receptors; HMGB1, High mobility group box 1; AHR, Aryl hydrocarbon receptor; CCR2, Chemokine receptor 2; MCP-1, Monocyte chemotactic protein 1; MCP-3, Monocyte chemotactic protein 3; TNF-α, Tumor necrosis factor-α; ASO, Atherosclerosis; TAO, Thromboangiitis obliterans; AAA, Abdominal aortic aneurysm*.

**Table 2:** Advantages and limitations of mice and rabbits in animal models of ASO.

**Species**	**Mice**	**Rabbits**	**References**
Advantages	Low cost (relatively low cost of animals, low cost of intervention research) Easy to obtain, breed and raise Space requirements for CSE interventions are relatively small Easy to genetic modification	Organizational availability is relatively large Easy to obtain, breed and raise Translational medicine research Relatively easy technical operation	(140–143)
Limitations	Less organizational availability Technical difficulties Wild-type relative anti-atherosclerosis	Relatively high costs (relatively high animal costs, high intervention research costs) Space requirements for cigarette smoke interventions are relatively large	
ASO	Animal models of genetic modification of multiple key genes (e.g. ApoE-/-, LDL-/-, etc.) Lipid metabolism is quite different from human ApoE -/- Fbn1 C1039G+/- mice can mimic human atherosclerotic plaque rupture and bleeding more closely	WHHL rabbits Unique lipid metabolism, much closer to human The process of atherosclerosis is closer to that of humans	(144–148)

*ASO, Arteriosclerosis; CSE, Cigarette smoke extract; WHHL, Watanabe heritable hyperlipidemic*.

## Smoking and Thromboangiitis Obliterans

### Clinical Evidence

Thromboangiitis obliterans (TAO), or Buerger's disease, is a group of unexplained non-atherosclerotic inflammatory diseases mainly caused by small and medium-sized arteriovenous vascular lesions. It is characterized by the involvement of vascular thrombosis (with or without recanalization), infiltration of inflammatory cells (in the tissue surrounding the thrombus or the adventitia), fibrosis of the intima and media, and changes in the internal elastic layer. Among people with a low socioeconomic status, smoking history is an important risk factor for this condition, and a strong correlation has been observed between heavy tobacco use and thromboembolic vasculitis (149, 150). At present, a definite diagnostic standard for diagnosing the disease is unavailable, but it mainly depends on the medical history, physical examination, laboratory examination, imaging examination and exclusion of other diagnoses. The simplest and most effective method to prevent further aggravation of this diseases is to stop smoking completely or use tobacco in any form (151, 152). After the diagnosis of TAO, patients who stop smoking have a lower risk of amputation than those who continue to smoke (153).

### Smoking-Related Molecular Mechanisms

Currently, although a large number of studies have confirmed that smoking is closely related to the occurrence and development of TAO, its specific mechanism is not yet clear. Under physiological or pathological conditions, the role of NO in maintaining vascular wall tension is mainly regulated by eNOS (154). However, the T allele of the eNOS 894 G-T polymorphism is associated with the prevention of TAO (155). Charles J. Glueck et al. also considered that mutations in the eNOS gene locus interact with cigarettes to reduce the production of vasodilatory NO and promote arterial spasm thrombosis. However, TAO has potential therapeutic value by promoting NO production and improving homocysteine metabolism (128). A pathological examination of the diseased vascular intima of patients with TAO revealed an immune response (156). Anticardiolipin antibody (ACA) is an autoantibody that uses negatively charged cardiolipin on the platelet and endothelial cell membrane as the target antigen and is commonly detected in patients with systemic lupus erythematosus and other autoimmune diseases (157). Clinical studies have confirmed that ACA may be related to Buerger's disease, which may exacerbate thrombotic events in patients with the disease (158). An analysis of clinical cases found that thromboembolic vasculitis was associated with an increased prevalence of anticardiolipin antibodies and that the presence of high antibody titers in these patients was associated with increased morbidity, including large limb amputation (156, 159). Previous reports have documented the correlation between smoking and anti-cardiolipin antibodies. As in-depth clinical research is conducted, researchers have proposed that smoking may cause physiological changes in some patients, resulting in the formation of aPL. Of course, this finding has also been confirmed in studies of many healthy individuals who later developed vascular disease (160, 161).

In addition, studies have shown that long-term cigarette exposure will lead to damage to the antioxidant defense system, which in turn increases the expression of MMP-9 and high mobility group box 1 (HMGB-1); high expression of MMP-9 and HMGB-1 also plays a role in TAO-related vascular lesions (136, 137). In summary, a large body of literature has established a link between smoking and TAO. However, the occurrence of TAO is not caused by a single factor. It is affected by a number of factors, including tobacco sensitization, cold, autoimmunity and changes in hormone levels. Therefore, clinical and molecular studies on TAO still must overcome numerous challenges.

### Smoking and Animal Models of TAO

The existing research on TAO mainly focuses on *in vitro* and clinical research. Although animal models of TAO (induced by sodium laurate) have been generated, the establishment and confirmation of these models are only confirmed by pathological examinations (162, 163). However, it still has its advantages and limitations *in vivo* animal models (162, 164) (Table 3). Due to our limited understanding of the pathogenesis of TAO, it is difficult to simulate the true pathogenesis of TAO *in vivo* (169, 170).

**Table 3:** Advantages and limitations of animal models of TAO and AAA.

**Disease**	**Model**	**Advantages**	**Limitations**	**References**
TAO	Sodium laurate	Good reproducibility It can simulate the symptoms and signs of TAO patients Pathological examination was consistent with TAO Combined tobacco smoke intervention is closer to the occurrence of TAO in humans	Morphology is in line with TAO performance, but it cannot fundamentally simulate human TAO Open surgery The technology is relatively difficult	(162, 164)
AAA	CaCl_2_, Elastase	Good reproducibility Applies to most species Rupture of AAA is common	Laparotomy Technical operation is difficult Increases the chance of abdominal infection	(165–168)
	AngII	Good reproducibility Laparotomy is not required	High cost The time to build the model is relatively long For mice only	

*TAO, Thromboangiitis obliterans; AAA, Abdominal aortic aneurysm; CaCl_2_, Calcium chloride; AngII, Angiotensin II*.

## Smoking and Aneurysm

### Clinical Evidence

Abdominal aortic aneurysm (AAA) is an abnormal enlargement of the infrarenal aorta. The pathophysiology of AAA causes the aortic wall to become increasingly thinner, which may rupture and cause life-threatening bleeding. Various factors may cause AAA. According to the classification of risk factors for AAA, smoking is the most important risk factor leading to aneurysm-related hospitalization and death, followed by the male sex and hypertension (171–173). A large number of retrospective studies, systematic reviews and other studies have shown that both active smoking and passive smoking may lead to the occurrence of aortic aneurysms and increase the risk of death. Women are often more sensitive than men. However, the occurrence of abdominal aortic aneurysms after quitting smoking is also strongly linked to a previous smoking history, but the risk of developing abdominal aortic aneurysms decreases faster in women than in men after quitting smoking (174–176). A study of the effect on smoking on small abdominal aortic aneurysms showed that continuous smoking is directly related to the prognosis of patients with small AAA, and patients with higher plasma cotinine concentrations (smokers) have an increased risk of rupture. The long-term survival rate is reduced (177).

In addition, smoking may exert a direct effect on perioperative and long-term complications after AAA. In terms of the effect of smoking on post-operative AAA, quitting smoking more than 8 weeks before opening and repairing AAA significantly reduces the incidence of post-operative pulmonary complications. However, elective open AAA repair after smoking cessation for <8 weeks has no correlation with the perioperative prognosis (178, 179). Although smoking cessation may reduce the risk of rapid progression of AAA, it still exerts a lasting effect on the development and operation of AAA, and the condition is more likely to occur in smokers than non-smokers. Therefore, the prohibition of smoking may effectively control the occurrence of AAA and the occurrence of serious complications (129).

### Smoking-Related Molecular Mechanisms

At present, the pathogenesis of AAA is mainly attributed to inflammatory cell infiltration, the destruction of elastin and collagen in the media and adventitia, biological changes in smooth muscle cells and angiogenesis (180, 181). Studies have confirmed that cigarette extracts induce the conversion of macrophages to osteoclasts through the classic RANKL-RANK pathway, upregulate the expression of NFATc1, promote macrophage activation, and induce the expression of the osteoclastogenesis-related proteases TRAP, cathepsin K and MMP-9 (129–131). Second, CSE also enhances the inflammatory response through the NF-kB pathway, which leads to the activation of macrophages and increased expression of MCP-1, MMP-9, IL-8, and TNF-α. These proinflammatory mediators and proteases are closely related to osteoclastogenesis and play a vital role in the formation of aneurysms (132, 133). Upon stimulation with cigarette smoke extracts, MMP-9 secreted by macrophages and MMP-2 secreted by mesenchymal cells cooperate to produce AAA. On the one hand, the mechanism is to activate TGF-β by cleaving its latent form, and TGF-β activation plays an important role in controlling tissue remodeling under physiological and pathological conditions. On the other hand, both enzymes inactivate protein-1α by cleaving serpins at an inhibitory site, and cleaved serpins induce the expression of proinflammatory molecules in monocytes (134, 135). Keisuke Hashimoto et al. added deoxyribonucleic acid (DNA) to the diet of mice to understand the effect of dietary DNA on blood vessel walls. An examination of the aorta revealed that dietary DNA may attenuate nicotine-induced elastic fiber degradation by inhibiting the MMP-2-dependent pathway, thereby preventing the weakened elasticity of the aortic wall (182). The role of MMPs in the formation and progression of AAA is indisputable (183, 184). The dialogue between cells (ECs, VSMCs, and macrophages) plays an important role in CSE-induced MMPs production in the aortic wall (135, 185), but its specific molecular mechanisms need to be further in-depth explored.

### Smoking and Animal Models of AAA

How does smoking affect animal models of AAA? At present, the three most commonly used animal models of AAA are calcium chloride-induced AAA, subrenal abdominal aortic perfusion with elastase, and subcutaneous injection of angiotensin II (AngII) (186, 187). Smoking not only affects the mechanical properties of the aorta and substantially changes the structure of the arterial wall but also changes gene expression through epigenetic mechanisms, thereby promoting the formation of AAA (188, 189). In a model of AAA induced by AngII, smoking further increased the expression of a matrix metalloproteinase (MMP) gene in the abdominal aorta, thereby enhancing the proteolytic activity of MMP (190). At the same time, 3,4-benzo pyrene in cigarette smoke also promotes the formation of AngII-induced AAA in mice through the same mechanism (191). However, in the model of AAA induced by elastase perfusion, the effect of smoking on the development of AAA does not depend on the activity of elastase, and smoking does not alter the expression of MMP-9 or MMP-12. However, smoking increases elastin degradation and the aneurysm size (192, 193). Of course, in order to simulate the aneurysm model more vividly, some special surgical treatments (such as aortic coarctation) on the basis of drug induction are often more effective in increasing the diameter of the aneurysm (194). However, AAA are often associated with arteriosclerosis factors. Therefore, establishing an animal model of AAA on the basis of arteriosclerosis (such as a high-fat diet) will be closer to the occurrence of human AAA (195). Although the existing AAA models do not fully replicate the pathological changes in AAA, the development of animal models has substantially improved our knowledge and understanding of the disease (165–168) (Table 3).

## Summary

With aging of the population, the incidence of PAD is increasing annually. Many factors promote the development of this disease, and smoking is an independent risk factor for PAD. The effect of tobacco exposure on PAD is continuous. Quitting smoking can partially reverse or delay disease progression, but it is not completely eliminated. As for the pathogenesis of smoking and PAD, *in vitro* studies, cigarettes and their substitutes can regulate the occurrence and development of PAD through a variety of pathways. *In vivo* studies, numerous studies confirm the association between smoking and cardiovascular disease. However, due to the advantages and limitations of different animal models, as well as the differences in the components of cigarette smoke and intervention techniques, the mechanism of smoking on cardiovascular diseases cannot completely mimic the pathogenesis of humans. Therefore, in the study of animal models of smoking and peripheral arterial disease, we should select appropriate animal models for related research based on the purpose of the research and combining the pathophysiological mechanisms of human diseases. At the same time, with the development of gene editing technology, we also look forward to the development of genetically modified animal models related to PAD, which can better simulate the occurrence and development of human PAD.

## Author Contributions

WW and TZ designed the study and searched literature. WW, KG, and GY contributed to drafting of the manuscript. All authors have read and approved the manuscript and agree to be accountable for all aspects of the research in ensuring that the accuracy and integrity of any part of the work are appropriately investigated and resolved.

## Conflict of Interest

The authors declare that the research was conducted in the absence of any commercial or financial relationships that could be construed as a potential conflict of interest.

## Publisher's Note

All claims expressed in this article are solely those of the authors and do not necessarily represent those of their affiliated organizations, or those of the publisher, the editors and the reviewers. Any product that may be evaluated in this article, or claim that may be made by its manufacturer, is not guaranteed or endorsed by the publisher.
